# Housing system reform: the opinion of advisory boards versus political reality in the Netherlands

**DOI:** 10.1007/s10901-012-9288-3

**Published:** 2012-05-10

**Authors:** Peter Boelhouwer, Hugo Priemus

**Affiliations:** OTB Research Institute for the Built Environment, Delft University of Technology, P.O. Box 5030, 2600 GA Delft, The Netherlands

**Keywords:** Housing policy reform, The Netherlands, Housing systems

## Abstract

This paper describes to what extent a more or less collective feeling of urgency to reform the Dutch housing market is addressed in the political arena. By doing that, it sheds some light on the effectiveness and influence of academic research and recommendations on the political decision making process. We conclude that the suggestion of several advisory bodies to start a serious reform of the housing system in the Netherlands is, due to coalition considerations, almost fully neglected by the Dutch Government. Although there is a common understanding among experts and interest organisations in the Netherlands that the current housing systems needs radical changes, coalition politics in the Netherlands are apparently more important to explain current housing policies. We conclude that the effectiveness and influence of academic research and recommendations on the political decision making process was quite modest in the last couple of years and try to explain the gap between academic research and political decision-making on Dutch housing policy.

## Introduction

There is growing consensus among experts, advisory bodies and many stakeholders in the Netherlands about the need to transform the housing system, in particular by a strategic reform of the housing finance system. This was the message of the Dutch Housing, Spatial Planning and Environment Council—generally abbreviated as VROMRaad in Dutch and VROM Council in English—(VROMRaad 2007) and, more recently, of the expert committee of the Dutch Social Economic Council—abbreviated as SER-CSED in Dutch but referred to in this paper simply as the CSED (SER-CSED 2010). The main arguments behind both proposals were recently laid out by Priemus (2010a, b). For an overview of the VROMRaad-proposals: see Boelhouwer and Hoekstra (2009). Advisory councils are very well rooted in Dutch society. In terms of the welfare state, the Dutch society can be characterized as a corporatist/social-democratic archetype. Negotiations and advise from societal parties are very well connected to such welfare states. The Dutch use the ancient word “polderen” to describe the sometimes endless negotiations between the organisations of civil society and government.

The purpose of this paper is to analyse to what extent this more or less collective feeling of urgency to reform the Dutch housing market is addressed in the political arena nowadays. By doing that, it sheds some light on the current effectiveness and influence of academic research and recommendations on the political decision making process. This theme is certainly not only relevant for Dutch readers. Due to globalisation and demographic, economic and cultural changes, many countries cope with housing systems which could be explained by their path dependence but are no longer well fitted to the actual changing world. Also many academics in for instance the European Network for Housing Research (ENHR) are involved in discussions in their countries about how to improve their sometimes badly functioning housing markets. An example from Britain are the many academics who were involved in the Barker report to improve the supply of new housing in the UK. This policy paper is not only a plain description of the different policy proposals presented by the advisory boards and political parties. It also gives a hopefully well-argued critical review based on the ideas and thoughts of both authors.

As mentioned above, since the Second World War, there were never so many proposals by academics, advisory bodies and stakeholders in the Netherlands to introduce policy changes in the housing system. To illustrate the perceived need to introduce fundamental changes, we firstly present an overview of the current CSED proposals for an integral reform of housing finance (Sect. 2). Then we confront these reform proposals with the election manifestos of the most influential political parties in the Netherlands. These proposals were written before the parliamentary elections held on 9 June 2010. The priorities of the various political parties in the field of housing as reflected in their election manifestos are reviewed in Sect. 3.

After these elections, the VVD (Conservatives), turned out to be the biggest political party, for the first time since 1945. It has 31 seats in the new parliament, just one more than PvdA (Labour). The biggest winner was the PVV (Party of Freedom, a new islamophobic political movement headed by Geert Wilders), which now has 27 seats, and the biggest loser was CDA (Christian Democrats), which saw its number of seats fall from 41 to 21. A minority coalition of VVD and CDA was formed, supported by PVV (without seats in the Cabinet). This support is officially formulated in a so-called Tolerance agreement between PVV, VVD and CDA).

Section 4 deals with the housing policy laid down in the new coalition agreement between VVD and CDA. We also compare these plans with the recommendations of the VROM Council and CSED for reforming the housing system and present some conclusions about the effect on the political process. The first author of this paper was chairperson of the VROM Council committee which prepared VROMRaad (2007), the second author was member of the SER-CESD committee, which published SER-CSED (2010). Are the proposals of the VROM Council and SER-CSED now at the heart of the political process? The answer is no, on the contrary they are neglected by politicians. In the final section we try to explain the differences between academic recommendations and current national housing policy in the Netherlands.

## Integral housing finance reform according to SER-CSED (2010)

The SER expert committee (CSED) chaired by Professor K. P. Goudswaard published its report entitled ‘Towards integral reform of the housing market’ on 16 April 2010 (SER-CSED 2010). Employers, employees and members of the SER appointed by the Crown have not expressed an opinion on this report. The CSED report constitutes an independent contribution to the discussion on fiscal policy concerning home ownership and social housing policy. This paper summarises this report of the SER expert committee.

### Accumulation of inconsistencies

The CSED states that the housing system in the Netherlands is an accumulation of inconsistencies, into which billions of euros are pumped around annually. The mortgage interest tax relief has stimulated borrowing in the home ownership sector, making the Netherlands the EU country with the highest relative mortgage debt: €640 billion. High-income households profit more from this support than low-income ones. The mortgage interest relief in combination with the low imputed rent has probably caused property prices to rise by about 20 % on average. The transfer tax (6 % of real estate price) functions as a tax on moving home, thus leading to welfare losses in housing and labour markets.

In the rental sector, housing allowances have been introduced to make rental accommodation more affordable for low-income groups. These allowances mainly support low-income groups. The difference between regulated and free-market rents can be regarded as an implicit subsidy for the tenants concerned. The rents of 95 % of rental properties are regulated. In 2008, the Dutch Bureau for Economic Policy Analysis (CPB) calculated the level of free-market rents (Romijn and Besseling 2008). Assuming a net yield of 5.7 %, the authors conclude that the free-market rents are much higher than the maximum reasonable level, determined by Dutch rent regulation. In consequence, the CPB concludes that it is primarily middle-income groups that profit from the indirect subsidies in the rental sector. These CPB calculations have been widely criticised (Priemus 2008; Boelhouwer 2008). The CPB later suggested that the yield for commercial real estate investors might be 1 %-point lower, making free-market rents lower than they had suggested previously (Donders et al. 2010). The truth is that we do not know the level of free-market rental prices, partly because we do not know how the market will respond to rent increases over a number of years. It is certain, however, that there are marked regional variations in free-market rents. Whether demand is likely to fall, and if so to what extent, will depend mainly on the approach taken to housing allowances. These allowances can prevent, at least reduce a drop out of demand.

There are finally questions about the position of housing associations—their performance, core tasks, whether there is a level playing field in their competition with commercial landlords, and their efficiency (Koning and Van Leuvensteijn 2010).

In any case, the inconsistent measures the government has taken have caused the gap between renting and buying to increase and mobility to fall; moreover, government has been pre-determining consumer attitudes to home ownership, implicitly promoting property ownership for high-income households and renting for low-income households.

### Future vision and transition policy

The CSED proposes a total overhaul of the country’s housing policy. It starts by sketching a final picture that could be reached around 2040, and then gives an outline of the transition period encompassing the 25 years from 2016 to 2040. The critical period from 2011 to 2015, a period with a weak housing market, should, according to the CSED, be used to ensure that all legal requirements are met after a social and political consensus has been reached about the proposed reforms. Because the transition period will span many different coalition governments, a broad political basis for the policy reform is urgently required.

The CSED’s vision for the year 2040 is divided into three components: the home ownership market, the rental market and housing associations.

### Future vision on the home ownership market

The CSED distinguishes four variants for the future fiscal treatment of home ownership. These are summarised in Table 1.

**Table 1:** Fiscal variants for home ownership according to the CSED

Current policy	Future policy			
	Own home as an investment asset (variant 1)	Own home as an investment asset, with a €200,000 tax exemption cap (variant 2)	Own home as consumer good (variant 3)	Own home removed from tax system (variant 4)
Relatively low flat-rate tax liability (max 0.55 %) on **capital invested in own home** and taxed at a marginal rate (15.55–52 %); placed in box 1 (income)	Standard flat-rate tax liability (4 %) of return on capital invested in own home, which is taxed at a fixed rate (30 %); placed in box 3 (capital)	As variant 1, but with a maximum tax-free allowance of €200,000; home owner receives tax refund in case of a negative tax	For home owners, no tax liability for their imputed rent	
Deduction of actually paid **interest on mortgage debt** which is taxed at a marginal rate (15.55–52 %) and placed in box 1 (income)	Standard flat-rate (4 % of mortgage debt), taxed at a fixed rate (30 %); placed in box 3 (capital)			No tax deduction for mortgage interest or debt

*Source*: SER-CSED 2010: 34

The CSED (2010: 37–38) states that the consumer-good and complete tax exemption (variants 3 and 4) are not attractive options for housing finance reform. Tax exemption (variant 4) has the disadvantage that it encourages people to invest in bricks and mortar, which is at variance with the general fiscal principle that the different forms of capital should be treated in the same way. The main objection to the consumer-good variant (variant 3) is that it subsidises home consumption across the board.

The investment asset variant (variant 1) has the important advantage that it no longer yields a tax benefit for living in one’s own home. This variant does however lead to much lower house prices and much higher housing costs. The extensive tax yield that would be released if the current tax regime were converted into the investment asset variant would have to be pumped back into the system to create a positive effect on purchasing power, according to the CSED. The advantages of the investment asset variant apply, albeit to a lesser extent, to the capped exemption variant (variant 2). The subsidy on living in one’s own home is limited in this case to €200,000. This variant thus offers better guarantees for low-income groups. It gives a fiscal advantage up to a mortgage debt of €200,000 (which would seem to be an appropriate level); above that sum, the advantage gradually tails off. In other words, home consumption is encouraged and is regarded as a ‘merit good’ up to a mortgage debt of €200,000. Variant 2 has less effect on house prices and housing costs than variant 1. As long as the housing market remains fragile, this can be considered as a major advantage of variant 2.

Although variant 1 represents by far the highest yield for the public budget, the CSED provisionally prefers the use of a capped exemption for owner-occupiers, as in variant 2, because of the vulnerability of the current housing market in the Netherlands. This means, that all home buyers and landlords receive substantial subsidies. The CSED however would like to work towards variant 1 in the long run, for example by abandoning the indexation of the tax exemption in the long term.

Furthermore, the CSED advocates phased abolition of the transfer tax and of tax exemption for the capital insurance on the sum invested in one’s own home.

### Future vision on the rental market

The CSED advocates a development in the direction of free-market rents, a regulated annual adaptation of rents and a tenure-neutral policy. This gives both housing associations and commercial real estate investors the best incentives to invest in rental properties. The current property valuation system would then be gradually phased out. In order to protect households with a modest income and other vulnerable groups, an additional housing allowance dependent on income would be introduced for both tenants and owner-occupiers. The amount payable would depend on income and household size, an important condition being that the household in question is the sole occupant of the home. This approach would counter rationing effect of equilibrium prices on housing demand. The housing allowance is intended to guarantee affordability and freedom of choice for the target group and to limit the risk of the poverty trap.

As the counterpart of the exemption up to €200,000 in the home ownership sector, the CSED advocates introduction of a (non-means-tested) *rent allowance* of up to €2,400 per year to ensure tenure neutrality of the housing policy.

### Future vision on housing associations

Once a rental policy with long-term regulation of rent adjustments, which is much more dynamic than the current inflation-linked rental policy, is announced, the value of the housing associations’ assets will rise sharply. It would be undesirable to leave decisions about the spending of this extra capital entirely up to individual housing association directors. The SER expert committee has sketched two scenarios to deal with this situation (SER-CSED 2010: 42–45): the Integral housing associations variant and the Split-function variant.

### Integral housing association variant

The *Integral housing association variant* involves the operation of a *Wet Bestemmingsheffing Vermogensovermaat Woningcorporaties* (Housing Association Excess Capital Levy for Housing Purposes Act), which ensures that about ninety per cent of the capital growth due to the introduction of a market-oriented rental policy is levied from each housing association in a single operation. The capital growth in question is the difference between the housing association’s operating value (*bedrijfswaarde*) under the old and the new rental policies. The levy does not have to be paid immediately in cash, but has the form of an obligation included in the balance sheet to make appropriate annual payments to a *Fonds voor de Volkshuisvesting* (Social Housing Fund), which still has to be set up. The annual payment is determined on the basis of the housing association’s expected cash flow, with the aid of data from the Central Social Housing Fund.

The ultimate value of the property owned by the housing association under market rental conditions will not be known at the time when the reforms are introduced. This means that the Housing Association Excess Capital Levy for Housing Purposes Act will have to embody means of modifying the amount of the overall commitment periodically. The CSED does give an indication of the sum transferred, however. Assuming an operating value of the social housing sector of 86.4 billion Euros in 2009, a future market value of the assets of the housing associations (in a rented state) of about 256 billion Euros and a yield of 4 per cent, the CSED estimates that the housing associations could make annual payments to the Social Housing Fund of the order of 6 billion Euros.

In our opinion, this amount is on the high side and may provisionally be taken as a long-term maximum estimate.

The increased operating value of the housing associations’ capital also represents capital, which should be used exclusively for social housing. The housing associations will be expected to prevent risk selection and to improve liveability from the yield derived from this capital, within the limits set by national policy. Finally, the housing associations are under an obligation to use their current operating value in the interests of social housing. Whenever this capital becomes liquid, for example through higher rent incomes and the sale of property, the vast majority of the proceeds must be transferred to the Social Housing Fund.

The advantage of this variant is that it fits in with the current structure of the housing associations, the proposals of the former Housing minister Van der Laan as laid down in letters he wrote to Parliament on 12 June 2009 and 15 December 2009 (*Kamerstuk* [Parliamentary document] 29453) and the agreement of December 15, 2009 between the cabinet and the European Commission on state support for housing associations. The risk is however that management problems will persist and the political vulnerability and administrative complexity will increase in this new construction. Both the obligations to the Fund and the annual payments will have to be subject to periodic political review.

### Split-function variant

In the *Split*-*function variant*, a division is created between the actual activities of the housing association and its capital. Most of the housing associations currently have the legal form of a foundation. In order to create a legally valid division between capital and management activities, the foundation will have to set up a new company to which all real activities of the housing association (ownership of the property, management and development) are transferred. The foundation then only holds the shares in this company. The new company acts as a regular commercial enterprise and strives to achieve good profits on the exploitation and development of the rental property. The profits are either distributed as dividend to the shareholder (=the foundation) or invested with the aim of increasing the profits in the future—which will once again go to the shareholder.

The original housing association (=the foundation) is thus converted into an investment fund and asset management firm; the only link between it and the company is that it holds the shares in the latter. It is governed by the provisions of the *Besluit beheer sociale huursector* (Social Rental Sector Management Order; Dutch abbreviation: BBSH). In accordance with the terms of this Order, surpluses are to be used in the interests of social housing in conformity with guidelines drawn up by Parliament. The obligation to use the housing association’s capital in the interests of social housing is thus also preserved in this variant. Assuming a capital of 256 billion Euros and a real yield of 4 per cent per annum, the CSED calculates that this would generate a contribution of about 10 billion Euros per annum, part of which would be reinvested. It would be convenient if the investment funds (foundations) could make use of the infrastructure of the inland revenue to make the necessary payments.

The shares in the company are in principle freely tradable. The funds (foundations) will sell part (preferably the greater part) of their shares in the company and invest the yield in other equities. This thus creates an economic as well as a legal separation between activities and capital. The economic separation has the advantage that an external shareholder can exert more effective pressure on the management to operate efficiently. The new shareholders will receive their share of the company’s dividends. Pension funds are typically likely to be interested in acquiring the shares, as long as the relationship between yield and risk is favourable.

Since there are benefits of scale in asset management, it is conceivable that the funds (foundations) might be interested in making use of one or more common asset management companies.

In this variant, the capital of the funds (foundations) is valued on the basis of the stock-exchange valuation of the shares in the company and the other equities held by the funds (foundations). This has the advantage of avoiding political or administrative conflicts about the best way to value the assets in question.

The more liquid nature of the capital will increase the temptation to squander the assets. Another potential problem is that the politicians will have to relinquish their discretionary control of rental policy in order to attract new potential shareholders.

Table 2 summarises the differences between these two variants and provides an answer to the following questions:

**Table 2:** Two variants of reformed housing association sector proposed by CSED

	Integral housing association variant	Split-function variant
Method used to activate capital	Levy on about 90 % of capital growth, to be paid in annual instalments	Annual payment of yield on capital owned by fund (foundation)
Safeguards of public interests A: housing of lower-income groups B: prevention of risk selection C: liveability guarantee	A: Via housing allowance and restrictions on housing association’s target group (households with incomes up to €33,000, 90 % of property assigned to target group) and municipal regulations concerning mixed housing development B: Via a duty to accept vulnerable groups C: On basis of performance agreement with municipality	A: Via housing allowance and municipal regulations concerning mixed housing development B: Via commissioning by municipality C: Via commissioning by municipality
Responsibility for transparency of objectives and governance	Central government via (replacement of) BBSH and (successor to) CFV, municipalities via municipal and/or regional housing visions and performance agreements	Fund (foundation): central government via BBSH Operating company: external shareholders via wish to maximise profits

- How is the ability of the housing association to pay housing allowances and rent allowances from its capital guaranteed?
- Who is responsible for safeguarding the public interests, and how?
- Who ensures transparency of the objectives and governance, and how?

Various possible ways of reforming the housing association sector are conceivable, of which the CSED has described two as indicated above. In both variants, the capital or the yield on the capital is placed in a separate fund or funds to finance assistance to lower-income groups to help them to meet their housing needs. Whichever variant is chosen, the financial contribution from the funds must be enough to finance the services proposed by the CSED.

### Preference for integral housing association variant

The CSED does not make any choice between the two housing association variants. We do however make a choice. We can only interpret the split-function variant as a prelude to the complete transformation of the housing association as a non-profit organisation with a hybrid character, where the orientation towards a target group and the obligation to devote its capital to a specific (social) purpose in conformity with the Housing Act remain essential components, to a fully fledged commercial market player where the interests of the shareholders will in the long run prevail over the dedication to a particular target group and the obligation to use the funds for a particular purpose. In our opinion, this comes down to throwing the baby away with the bathwater. Housing associations have in the past made an essential contribution to maintaining social housing in the Netherlands at a proper level. They can continue to play this role if certain conditions are met. We are therefore convinced that the integral housing association variant is to be preferred over the split-function variant.

## Housing policy in manifestos of Dutch political parties in the Spring of 2010

The Dutch political parties published their (draft) election manifestos in the Spring of 2010. The statements on housing policy contained in these manifestos are reviewed in the present section (see Boelhouwer and Priemus 2010). The parties concerned are the CDA (Christian Democrats), PvdA (Dutch Labour party), VVD (Conservatives), D’66 (Social Liberals), *ChristenUnie* (a Protestant confessional party), *Groen Links* (the Dutch green party), SP (the Socialist Party) and the highly xenophobic PVV.

### Rental sector

Many political parties want to bring rents more in line with the market, even in the sector governed by housing associations. Many give priority to reduction of the mismatch of the rental market (high income tenants living in cheap social housing). The SP wants to extend the social rental sector to include rents of up to €850 per month, and is the only party to advocate prolongation of the inflation-linked rent policy. This proposal, if accepted, would make investment in both social and commercial rental accommodation much more difficult.

CDA, PvdA, D’66 (left wing liberals), *ChristenUnie (protestant party)* and *Groen Links* (left wing green party) want to make the rental policy more market-oriented by incorporating housing value assessment in accordance with the *Wet waardering onroerende zaken* (Real Property Assessment Act; Dutch abbreviation WOZ)—this assessment is generally known simply as the ‘WOZ value’ in the Dutch context—in the points system used to determine maximum rental levels known as the *woningwaarderingsstelsel* (dwelling assessment system; Dutch abbreviation: wws) and by introducing a periodic means test: anyone found not to belong to the target group will then have to pay more rent for accommodation provided by a housing association, or will have to buy their home or can decide to move house to commercial rented housing, or an owner-occupied dwelling. These parties want the housing associations to concentrate more on their core tasks. Many political parties like the idea of using the increased value of the housing stock owned by housing associations (due to a more dynamic rental policy) to finance the housing allowance. Some parties advocate abolition of the Vogelaar levy (subsidies from richer housing associations to support investments in 40 selected urban problem districts) while the PvdA and SP would like to see imposition of a levy on rich housing associations to support the poorer ones. The PVV goes further, and advocates the introduction of a general levy on the assets of all housing associations. It claims that this would stimulate the sale of property owned by housing associations. *Groen Links* has the same aim, but would like to achieve this by giving all tenants of property owned by housing associations the right to buy their home. D’66 sketches another strategy for reducing the social rental sector: the introduction of an exit option for housing associations after they have transferred their capital gains to the Crown. The VVD would also like to see the housing associations sell part of their housing stock, but does not specify any extra instrument to this end. In fact, the VVD launches the strongest frontal attack on the housing associations, proposing that all rents should be liberalised and that the predicate ‘social rental housing’ should be abolished. Housing allowances should be used to make accommodation affordable; there would seem to be no room at all for housing associations in this vision.

### Home ownership

CDA, VVD and *ChristenUnie* are explicit in their advocacy of stimulation of home ownership in general. This does not however stop the *ChristenUnie* from wanting to tinker with the mortgage interest tax relief (*hypotheekrenteaftrek*; Dutch abbreviation HRA) by proposing that the tax deduction should facilitate paying off the mortgage by assuming (real or hypothetical) repayment on an annuity basis. They also propose capping the tax-deductible mortgage debt at €750,000 and reducing the maximum tax rate to 42 %. VVD and PVV do not intend to interfere with the mortgage interest tax relief. CDA adopts a striking formulation: ‘As long as the mortgage interest allowance stimulates home ownership and mobility on the housing market, it must be regarded as a valued instrument’. CDA leader Jan Peter Balkenende went so far as to state during the election campaign that he would not be prepared to work together in a coalition with parties that wish to interfere with the mortgage interest tax relief.

SP states that the mortgage interest tax relief should be guaranteed, but nevertheless proposed significant modification of this arrangement by stipulating that the maximum deductible debt should be €350,000 and the maximum tax rate 42 %. The SP also wishes to encourage repayment of the mortgage debt.

VVD wants to leave the mortgage interest tax relief unchanged but also wants to abolish transfer tax, putting a ceiling to the property tax. In addition, VVD wants to introduce a reduced rate of VAT for major maintenance and home improvement. CDA would also like to take tax measures to facilitate structural home improvement.

D’66, PvdA and *Groen Links* advocate reform of the fiscal consequences of home ownership—but in all cases with a long transition period. These parties would like to see a maximum tax rate of 30 %, measures to stimulate the repayment of mortgage debt and phasing out of transfer tax and imputed rent as part of one’s taxable income. *Groen Links* goes furthest in proposing measures to cover the financial risks of home owners who are faced by financial problems: according to this party, the authorities should buy up the dwelling or the mortgage in such cases.

We have the following comments on the election manifestos of the parties reviewed.

1. Only some of the political parties, in particular those on the left of the political spectrum, are in favour of an integral reform of the housing market. CDA and VVD in particular opt for reinforcement of the current imbalances on the housing market. These parties advocate major increases in the burden on tenants in the rental sector, while the home ownership sector is spared and even stimulated through tax cuts. PVV wants to keep things largely as they are. SP concentrates the proposed reform on the home ownership sector. As a result of these choices, no attention is paid to the integral reform of the housing market proposed in many housing market studies. Some proposals of the political parties would lead to an increase in the existing imbalances, and thus to a greater risk of market failure and government failure. An additional factor is that, under pressure from the need to cut the budget deficit, most political parties were already thinking of major cost cuts and heavier burdens on the players in the housing sector even before the negotiations leading to the formation of the next cabinet started. This is at variance with the advice of both the VROM Council (VROMRaad 2010) and the CSED (SER-CSED 2010). These two advisory bodies both advise politicians not to disrupt the weak housing market in the coming years. The economic crisis on the housing market will probably last longer than the general economic recession. On the other hand, a clear picture of housing market policy for the coming decades will have to be worked out during the next 4 years, thus removing the present uncertainty that has a paralysing effect on households’ willingness to purchase. This will allow the necessary cuts in government expenditure to be made without too many problems during the coming 10–20 years.
2. The various political parties emphasise that drastic cuts will have to be made in the housing budget, but they fail to make the fundamental policy choices needed in this connection. It will have to be made clear, for example, what minimum basic quality will and can be made available for the various income groups, and what level of spatial integration will be the political standard. The spatial framework has to be specified within which the extra housing stock required in the future should be built. Should we follow the recent recommendations from the OECD (OECD 2010) that spatial planning in the Netherlands should be strongly deregulated, or is it better to stick to the current compact city policy which will mean that the housing stock can only be expanded at very high cost? The proposed increase in the financial burdens on the general population will improve the relative market position of the less expensive housing stock. In particular, there will be an increased demand for dwellings that are currently earmarked for demolition. This means in its turn that the intentions of policy-makers on restructuring will have to be recalibrated.
3. The politicians will also have to lay down the necessary affordability standards. These policy parameters will have to be established before the reform proposals described above can be worked out and implemented, and a concrete value can be assigned to the tenure-neutral housing allowance proposed by CSED and VROM Council.
4. It is noteworthy that practically all political parties (with the exception of SP) opted to raise the burden on tenants substantially. This is particularly striking in view of the high level of expenditure with which Dutch tenants are currently faced. Tenants pay an average of 23 % of their disposable income on housing, which is much more than the 16 % spent by owner-occupiers, while the latter with their significantly higher income could more easily afford to spend more on housing than tenants (Bleije et al. 2010). It should be further noted that the level of housing expenditure in the home ownership sector is misleading since it does not take the interest on the invested capital into account. In fact, total housing-related expenditure for tenants in the social rental sector already amounts to some 38 % of their income on average while they are likely to be faced with increased expenses in the years to come due to the scarcity of fossil fuels even without taking rent rises into consideration. Recent data from the European Commission support this picture (Orsolya and Zolyomi 2009). European Commission Research Note No.1 (Housing and Social Inclusion) indicates that the Netherlands has the highest net expenditure on housing of the 25 EU Member States studied (30.9 % compared with the EU average of 22.2 %). When the most vulnerable households from the first income quartile are considered, the Netherlands has the second highest net expenditure on housing after Greece (47.4 % for the Netherlands compared with the EU average of 37.4 %). These results place the proposals of most political parties to raise the rents in a dubious light. Apart from the question of whether it is feasible to charge even higher rents in view of the large rental expenses tenants are already faced with, the implementation of such a policy would place the Netherlands in an even more eccentric position in a European context than it already is.
5. Practically all political parties believe that it would be highly beneficial to reduce the mismatch in the rental sector (the number of households living in rented dwellings that are cheaper than what they could reasonably afford). No one has anything to say about the comparable trend in the highly subsidised owner-occupier sector. For example, PvdA stated just before the elections that they intended to increase the expenses of tenants with an income exceeding 40,000 Euros per annum by 2.8 billion Euros. This proposal is unrealistic in our opinion, since very few households in the social rental sector pay a low proportion of their income on housing (Boelhouwer 2007). About 71 % of tenants in the social rental sector have an annual income of less than 33,000 Euros. This group spends an average of 40 % of their income on housing. The comparable figure for tenants with an annual income of up to 51,000 Euros fluctuates around 25 %. It is true that the small group with really high incomes (more than 51,000 Euros) in the social rental sector could afford to pay higher rents. The potential yield of such a measure would amount to some hundreds of millions of Euros rather than the billions of Euros mentioned in some election manifestos. It is against this background that the VROM Council (VROMRaad 2010) argues against developing any specific policy to reduce the so-called mismatch in the rental sector. The administrative costs would outweigh the possible gains. The political consequences of the mismatch could be dealt with by a gradual increase in rents and by fixing the rents more in conformity with market requirements (better relation between price and quality). When rents are in line with market requirements, everyone can choose the quality, location and rent level he or she prefers, given the restrictions on supply. In such a situation, there is no mismatch at all.
  It should be remembered that Dutch politicians have always expressed a political preference for creating ‘mixed’ neighbourhoods. Billions of Euros have been spent on urban restructuring needed to achieve this end. There is no justification for making the combating of a mismatch in the rental sector a major political target. The gains achieved in this way would be outweighed by the costs of creating mixed neighbourhoods.

No political party in the Netherlands has completely incorporated the plans put forward by CSED and VROMraad in its election manifesto, and no political party thinks 30 years ahead in its election manifesto. Several components of these proposals are included in the election manifestos. But in this way the integral reform, as proposed by VROMRaad and SER-CSED, will not be realised. However, the individual manifestos do seem to provide a basis for a fruitful compromise. Some parties focus mainly on the home ownership sector in their attempts to reform the housing market, while others concentrate on the rental sector. The announcement of CDA that the mortgage interest allowance system will be left unchanged during the next 4 years could also fit in the framework of a political compromise based on the proposals of the CSED and the VROMRaad.

## Housing policy in the coalition agreement (2010): freedom and responsibility

In comparison with the sometimes interesting and lively debates before the general elections, the attention housing gets in the government agreement “Freedom and responsibility”, concluded in October 2011, is rather limited. No coherent reform program is presented and the proposals lack a clear structure. There is also a big difference with the sometimes extensive policy proposals from the policy manifestos of the political parties. It is quite clear that the coalition government was not able or willing to introduce some really radical housing policy changes.

It is striking that the reforms and proposed budget cuts are mostly focussed on the (social) rented sector. The home ownership sector is almost not mentioned in the agreement and the huge fiscal subsidies to home owners are not discussed at all. This choice is in conflict with all earlier discussed advices and also with most of the election manifestos. All advisory bodies advocated an integral reform of the housing system, including both renting and owning.

The coalition parties define a reform of the rental market, but are silent about a reform in the owner-occupied market.

In the rented sector, the policy of the yearly rent adjustment by inflation is continued, against all recommendations. Most important reason for the government to not introduce a more market oriented rent adjustment system is that such a policy would lead to negative effects on the purchasing power of tenants. Only renters with an income of above 43.000 euros per year will be confronted with a rent increase of 5 % plus inflation (at this moment almost 2 % in the Netherlands). This strong rent increase will, however, only effect about 15 % of the total households in the rented sector. It is also still a question if this proposal can be implemented. Tax authorities are the only institution who can check income data. They are already complaining about an overload of commitments. Checking the incomes of 2.4 households in social housing is costly, time consuming and sensitive to fraud.

The coalition agreement has most far reaching consequences for the social rented sector in the Netherlands. First of all tenants in the social rented sector, after a similar policy in Great Britain since 1980 receive a right to buy. This proposal is introduced to stimulate home ownership furthermore and to compensate the landlords for a big property tax which will be introduced in 2014 and has to raise 760 million euros every year (620 million euros for housing associations; 140 million euros for commercial real estate investors). Government wants that landlords will pay part of the costs on housing allowances. The idea to introduce a right to buy is quite new and was not discussed before the election. By now the government is quite hesitating about this firm statement. It is obvious that housing associations in the Netherlands are private organisations which cannot be expropriated without compensation (Boelhouwer 2007). So in the opinion of all experts this proposal will not be implemented at all.

A proposal which is more in line with most of the advices and is ordered by the EU is that housing associations should allocate at least 90 % of their vacant dwellings to households with a maximum income of 33.000 euros (price level 1-1-2010; approximately 76 % of the current allocations).

## Tensions between the academic and the political domain: some observations

Many members from the advisory councils had an academic background. The members of the SER were all economists from academia and more than half of the members of the VROM council were also academics from different disciplines. These academics operate in scientific settings governed by empirical evidence and logic. Politicians, on the other hand, operate in public and political settings governed by majority voting. On the one hand, a productive relationship between the academic and the political domain is crucially important in order to develop evidence-based policy. On the other, a sharp distinction needs to be drawn between science and policy. Academics can carefully trace the past and define the present, but their normative statements on the desired policy seldom go uncontested: or, to put it bluntly, it soon emerges that there is more than one way to skin a cat. Academics should not take on the role of politicians, not even if there is consensus on the course that should be charted.

Politicians may have perfectly legitimate reasons for deviating from the recommendations of the academics. For example:

1. Academics usually work in a relatively limited field, but politicians have other things to think about, not least, the non-housing impacts of housing policies (Bourne 1981), juggling the claims in different policy sectors, the tax burden, and degrees of freedom for individuals and businesses. In the academic domain theorists try to extend the scope of systems, but scientific analyses still tend to be more limited in scope than political decision-making.
2. Another important factor is that political decision-making is driven by multiple interests, electoral considerations and, of course, the term of office (4 years), while academics generally reach conclusions in terms of public expenditure and cost-benefit analyses and thereby marginalise the multiple interests and push the time horizon far beyond 4 years.
3. A third difference is that academic decision-making usually takes a long time whereas political decision-making is invariably subject to tight schedules, particularly during the formation of a new Cabinet. These tight schedules, coupled with the fact that only a few people are involved in the process, create a strong probability that much relevant knowledge will be neglected during the political decision-making.
4. Finally, political decision-making is often a question of horse-trading—a phenomenon that is foreign to academic circles. Coalitions cannot be formed without compromises: parties concede ground in some policy fields and win it in others.

Alongside these general differences between academic and political decision-making, certain specific differences exist in the case that a housing finance regime will be considered:

1. It often takes a long time for academic findings to filter through to political decision-making. The timing of the SER-CSED report (2010) was particularly inopportune as it appeared after the political parties had drawn up their election programmes and after the elections to the House of Representatives—too late for incorporation in the decision-making on the coalition.
2. The coalition formation in 2010 was dominated by a necessity perceived by economists (also academics) to hack into public spending. The VROMRaad published its recommendations (2007) before the credit crunch unfolded and before the need to cut public expenses became increasingly urgent. The report by the SER Commission (SER/CSED 2010) did not anticipate fundamental cuts in government spending. The politicians cannot be blamed for reaching a coalition agreement based largely on austerity.
3. Both the VROMRaad (2007) and SER-CSED (2010) envisage a transition period of between 20 and 25 years. Coalition agreements cover a period of 4 years and are not regarded as policy documents for the decades still to come.
4. Politicians and academics agree that the Dutch construction industry and the housing market were in a sorry state in 2010 and that the prospects for the coming years were poor. SER-CSED (2010) states explicitly that the actual reforms to housing finance reform should not begin before 2015. Politicians realise that anticipating reforms in 2010 or 2011 that will not be introduced until at least 2015 will send out certain signals. Then a new cabinet will have made its start. House prices could already decline (more than they do now) if the public and the banks were under the impression that fundamental reductions in the mortgage interest relief were pending from 2015. Unlike academic debates, political debates can trigger repercussions in society and the housing market.
5. In the past 10 years home ownership in the Netherlands has passed the magic 50 % mark. At the moment 58 % of the housing stock consists of owner-occupied dwellings. What this amounts to in political terms is that owner-occupiers are now more important electorally than tenants. Many observers would see it as political suicide if any party were to pledge to phase out mortgage interest relief. That would be a step too far for a political party in such economically tough times.

As far as the future is concerned, it is good for academics to keep a finger on the pulse and show that the housing market problems will not just fade away. Reports by the IMF and OECD which urge the Dutch authorities year after year to reform housing market policy can step up the pressure on politicians. It is also vitally important for interest groups such as Nederlandse Woonbond (national tenants’ association), Vereniging Eigen Huis (national association of home owners), the AEDES association of housing associations, the Association of Dutch Municipalities, the Dutch Construction and Infrastructure Federation, NEPROM association of Dutch property developers and the IVBN association of Dutch property investors to get together and form a united front.

Last but not least, it would be helpful if the political parties were to work towards shared perspectives instead of adopting tight individual standpoints. Various coalitions will, after all, be needed to realise the proposed housing finance reform. A fifty-one percent majority is not enough to support it; what is needed is a broad cross-party platform. In the Netherlands further decision-making in the Social and Economic Council can help to build a broad public and political support base with the United Kingdom as a benchmark. In the 1990s the Conservative government in the UK started a housing finance reform (abolishment of mortgage interest tax deduction) that was continued by a subsequent government with the support of the Labour Party. Experience from Sweden and—to a greater extent—from the United Kingdom (Boelhouwer and Van der Heijden 1992) shows that housing finance reforms, like the ones proposed in the Netherlands, are politically and socially attainable.

## Conclusions

As mentioned in the last section, the current VVD-CDA government was not willing or able to introduce a serious housing market reform, based on the advices of the VROM council and the SER’s committee of socio-economic experts (CSED). The coalition agreement is even less integrated and ad hoc than most of the election manifestos of the political parties. A substantial proposal is a new property tax for landlords. As a result of this proposal, the misbalance between the government support of the rented and owner occupied sector will even be enlarged in favour of the latter. The introduction of the right to buy will due to property legislation at the end probably not been introduced. There are several reasons for this rather disappointing government agreement. One reason is that the government is afraid to deepen the economic crisis even more. A second reason is that there are big political differences on socio-economic policies between the conservatives, the christian democrats and the PVV. The latter party has a program that has much more in common with the socialist parties than with both coalition parties. For the last two parties it is not favourable to decrease the huge subsidies to owner occupiers and for the PVV low income groups in the rented sector are quite important.

On the basis of these results we can conclude that the suggestion of many advisory bodies to start a serious reform of the housing system in the Netherlands is due to coalition considerations almost fully neglected by the new government. Although there is a common understanding among experts and interest organisations in the Netherlands that the current housing systems needs radical changes, coalition politics in the Netherlands are apparently more important to explain housing policies. We can conclude that the effectiveness and influence of academic research and advice on the political decision making process was quite modest in the last couple of years. Most stakeholders are heavily disappointed with this outcome. On the basis of the mentioned advisory reports, they are now working on a common vision how to reform the Dutch housing market. It remains to be seen whether they will be more successful in determining the political agenda in the near future.

Against our call for long term coalition agreements could be argued that we are too dedicated to the school of policy analysis and to problem-orientated policy research. If we for instance take a closer look at the increasing bifurcation of Dutch politics in general and housing policy more specifically (the full unconditional support for home ownership by conservatives and the support for the civil society by christian and social democrats) it is easier to understand why these fundamental policy transformations are so hardly to introduce. Also the welfare state literature gives some clues. For instance Hacker (2002) argues that path dependency forecloses functionalist explanations that view policy configurations as optimally tailored to current conditions by politicians. By studying path dependency one gets to learn how contingent our institutions really are. Phenomena such as welfare states are characterized in an almost evolutionary manner, by complex traits fashioned at different times and in different ways in the course of their development (see for an overview of the housing sector in Europe also Boelhouwer and Van der Heijden 1992).
